# Anti-neoplastic Effect of Ginkgolide C through Modulating c-Met Phosphorylation in Hepatocellular Carcinoma Cells

**DOI:** 10.3390/ijms21218303

**Published:** 2020-11-05

**Authors:** Min Hee Yang, Seung Ho Baek, Jae-Young Um, Kwang Seok Ahn

**Affiliations:** 1 KHU-KIST Department of Converging Science and Technology, Kyung Hee University, Seoul 02447, Korea; didmini@naver.com; 2 Department of Science in Korean Medicine, Kyung Hee University, 24 Kyungheedae-ro, Dongdaemun-gu, Seoul 02447, Korea; jyum@khu.ac.kr; 3 College of Korean Medicine, Dongguk University, 32 Dongguk-ro, Ilsandong-gu, Goyang-si, Gyeonggi-do 10326, Korea; baekone99@gmail.com

**Keywords:** Ginkgolide C, HGF, c-Met, hepatocellular carcinoma cells

## Abstract

Ginkgolide C (GGC) derived from *Ginkgo biloba*, has been reported to exhibit various biological functions. However, the anti-neoplastic effect of GGC and its mechanisms in liver cancer have not been studied previously. Hepatocyte growth factor (HGF)/c-mesenchymal–epithelial transition receptor (c-Met) pathway can regulate tumor growth and metastasis in hepatocellular carcinoma (HCC) cells. This study aimed to evaluate the anti-neoplastic effect of GGC against HCC cells and we observed that GGC inhibited HGF-induced c-Met and c-Met downstream oncogenic pathways, such as PI3K/Akt/mTOR and MEK/ERK. In addition, GGC also suppressed the proliferation of expression of diverse tumorigenic proteins (Bcl-2, Bcl-xL, Survivin, IAP-1, IAP-2, Cyclin D1, and COX-2) and induced apoptosis. Interestingly, the silencing of c-Met by small interfering RNA (siRNA) mitigated c-Met expression and enhanced GGC-induced apoptosis. Moreover, it was noted that GGC also significantly reduced the invasion and migration of HCC cells. Overall, the data clearly demonstrate that GGC exerts its anti-neoplastic activity through modulating c-Met phosphorylation and may be used as an effective therapy against HCC.

## 1. Introduction

Hepatocellular carcinoma (HCC) is a lethal malignancy and third leading cause of mortality among cancer patients in the world [1,2,3,4]. Early-stage HCC patients can be treated with liver resection. However, most patients are diagnosed with advanced stages due to difficulty in early diagnosis [1,5,6,7,8,9]. Despite significant improvements in the availability of conventional therapies in recent years, the five-year survival rate of HCC is still poor at approximately 15% [10,11,12,13]. Thus, understanding the detailed molecular mechanisms of HCC progression and the identification of novel therapeutic targets can be a useful strategy for HCC management.

c-Met is a receptor tyrosine kinase (RTK) that can be activated by hepatocyte growth factor (HGF) [14]. The stimulation of c-Met activity leads to the rapid phosphorylation of downstream signals, such as RAS, MAPK, PI3K, and Akt, that can regulate cell growth, metastasis, survival, and motility [15,16,17,18,19,20]. Aberrant c-Met activation has been noticed in several human cancers including liver, colon, lung, breast, ovarian and gastric cancers [21,22]. HGF/c-Met elevation have been associated with metastatic progression in many major human cancers [22,23]. The poor prognosis of HCC is due to frequent recurrence and metastasis [24,25]. Thus c-Met can be considered as a vital therapeutic target for HCC therapy [24,25,26,27]. Interestingly, Gao et al. found that a common polyphenol, resveratrol, can attenuate HCC growth through targeting the HGF-c-Met signaling pathway, thereby further implicating the possibility that c-Met can be used as a suitable molecular target for prevention and clinical treatment of HCC [10].

Natural products remain a cornerstone for cancer prevention and treatment [28,29,30,31,32]. *Ginkgo biloba* (Ginkgoaceae) is an ancient Chinese tree which has been widely used as a medicinal herb [33]. The leaf extract of *Ginkgo biloba* has been employed for management of multiple disease such as cerebrovascular and cardiovascular diseases [34]. Also, previous reports found that *Ginkgo Biloba* extract inhibits invasion and metastasis by suppressing the ERK/NF-κB signaling pathway in gastric cancer [35]. Several compounds have been isolated from *Ginko biloba*, including diterpene lactones, pholyphenols, and flavonoids [36,37]. The ginkgo diterpene lactones mainly consist of bilobailde, ginkgolide A, ginkgolide B, and ginkgolide C [38]. They share a common twenty-carbon molecules and contain a six 5-membered rings [37,38]. Ginkoglide B could enhance gemcitabine sensitivity in pancreatic cancer cells through PAFR/NF-κB signaling pathway [39]. Wang et al. also found that Ginkgolide B had an anti-tumor effect via beclin-1-dependent autophagy in lung cancer [40]. Among the various bioactive components, it has been reported that Ginkgolide C (GGC) can effectively attenuate lipid accumulation and promote lipolysis through stimulating the Sirt1/AMPK pathway in oleic acid-exposed fatty liver cells [41]. It has also been found to increase lipolysis and suppress adipogenesis in adipocytes through regulating the AMPK pathway [42]. However, the potential anti-cancer effect of GGC in liver cancer has not been studied yet. c-Met has been reported to play an important role in various cancers, and it may be possible that GGC may exert its anti-cancer actions through modulating this oncogenic pathway. In our study, we determined whether GGC can induce substantial apoptosis through modulating c-Met cascades in human hepatocellular carcinoma cells.

## 2. Results

### 2.1. GGC Suppressed Cell Viability and the Phosphorylation of c-Met

**Figure 1:**
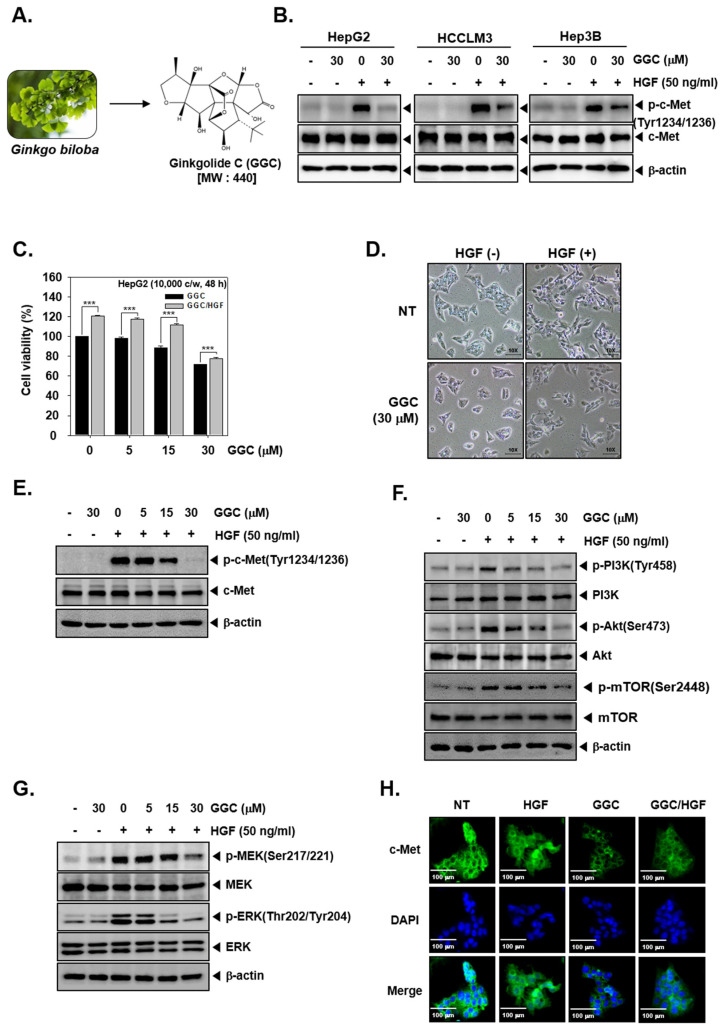
GGC suppresses HGF-induced Met phosphorylation. (**A**) *Ginkgo biloba* picture and chemical structure of Ginkgolide C (GGC). (**B**) HepG2, HCCLM3, and Hep3B cells (5 × 10^5^ cells/well) were pre-treated with GGC 30 μM for 2 h, then HGF (50 ng/mL) treated for 30 min and western blotting for p-cMet(Tyr1234/1236) and c-Met was done. (**C**) HepG2 cells (1 × 10^4^ cells/well) were pre-treated with (0, 5, 15, 30 μM) of GGC for 2 h, then HGF (50 ng/mL) treated for total 48 h and MTT assay was done. Data represent means ± SD. *** *p* < 0.001 compared to the control. (**D**) HepG2 cells (2 × 10^5^ cells/well) were seeded on 6 well plate. GGC (30 μM) was pre-treated for 2 h, then HGF (50 ng/mL) treated for total 24 h. Morphological changes were observed by Nikon ECLIPSE Ts2 (Nikon corporation, Japan). (**E**–**G**) HepG2 cells (5 × 10^5^ cells/well) were pre-treated with various concentrations of GGC for 2 h, then HGF (50 ng/mL) treated for 30 min and western blotting was done. (**H**) HepG2 cells were pre-treated with GGC (30 μM) for 2 h, then HGF (50 ng/mL) stimulated for 30 min and c-Met distribution was analyzed by immunocytochemistry (scale bar: 100 μm). The results shown are representative data of three independent experiments.

We analyzed if GGC could affect c-Met activation in hepatocellular carcinoma cells. As shown in Figure 1B, GGC negatively affected p-c-Met levels in HepG2, HCCLM3, and Hep3B HCC cells. To determine the viability, we used the MTT assay. HepG2 cells were pre-treated with GGC (30 μM) for 2 h and treated with HGF (50 ng/mL) for total 48 h. GGC suppressed cell viability in single treatment and also as GGC/HGF combination treatment. GGC and HGF co-treated cells displayed higher cell viability (Figure 1C). As shown in Figure 1D, HGF induced marked morphological changes into a spindle like shape and this alteration was reversed by GGC. We next investigated if GGC can also modulate the phosphorylation of c-Met and downstream signaling cascades. The phosphorylation of c-Met was only induced in HGF treated HepG2 cells and GGC significantly down-regulated c-Met phosphorylation, but there was no change in c-Met expression (Figure 1E). The phosphorylation of downstream signals, including PI3K, Akt, mTOR, MEK, and ERK kinases, was induced by HGF. GGC substantially down-regulated HGF-induced phosphorylation of downstream signals but there were no effects on the total PI3K, Akt, mTOR, MEK, and ERK expression (Figure 1F,G). Immunocytochemistry data further revealed that HGF induced c-Met (Figure 1H) and GGC treatment suppressed c-Met level as well as HGF-induced c-Met level.

### 2.2. GGC Inhibited Cell Proliferation and Promoted Apoptosis

**Figure 2:**
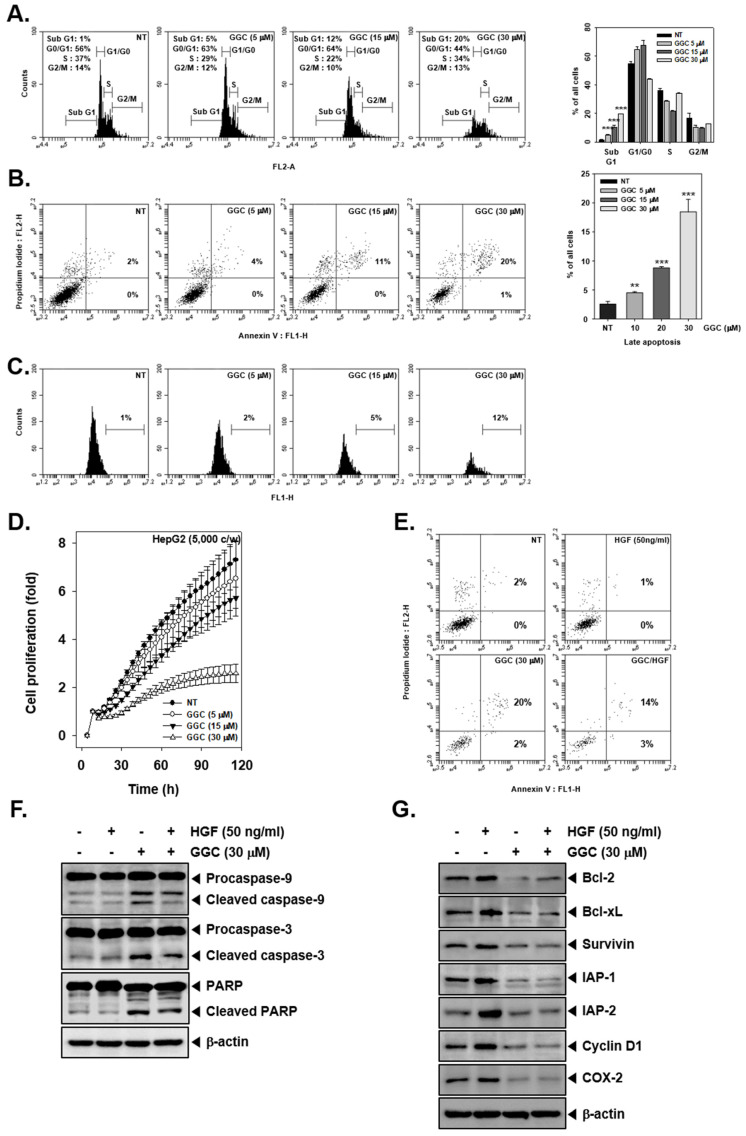
GGC inhibits cell proliferation and promotes apoptosis. HepG2 cells were treated with the indicated concentrations of GGC for 24 h (**A**) The cells were fixed in EtOH for overnight. RNase A (10 μg/mL) was treated for 1 h stained with PI and cell-cycle distribution was analyzed by flow cytometry. The results are presented as the mean ± SD. *** *p* <0.001 compared to the control. (**B**) The cells were stained with Annexin V FITC and PI for 15 min, then analyzed by flow cytometry. The results are presented as the mean ± SD. ** *p* <0.01, *** *p* <0.001 compared to the control. (**C**) The cells were fixed in 4% PFA (paraformaldehyde), incubated with TUNEL reaction solution and then analyzed by flow cytometry. (**D**) Cell proliferation was measured by using RTCA. (**F**,**G**) The levels of various proteins were analyzed by Western blotting. β-actin was used as an international control.

To analyze the apoptotic effect of GGC, we used various techniques such as cell cycle analysis, Annexin V assay, and TUNEL assay. As shown in Figure 2A, GGC induced sub G1 phase arrest in a dose-dependent fashion. Late apoptosis was also induced as the apoptosis increased with increasing drug concentrations (Figure 2B,C). In addition, GGC induced late apoptosis to a lesser extent in HGF treated cells (Figure 2E). We also noted that GGC reduced cell proliferation in HepG2 cells in a dose and time dependent manner (Figure 2D). Next, we investigated the expression of GGC-induced apoptotic markers by Western blot analysis. As shown in Figure 2F, GGC induced apoptosis through the cleavage of caspase-9, caspase-3, and PARP. HGF treatment reduced the activation of apoptosis markers and it also reduced HGF-induced the expression of various antiapoptotic proteins, such as Bcl-2, Bcl-xL, Survivin, IAP-1, IAP-2, Cyclin D1, and COX-2.

### 2.3. c-Met Gene Knock Down can the Activation of Downstream Targets

**Figure 3:**
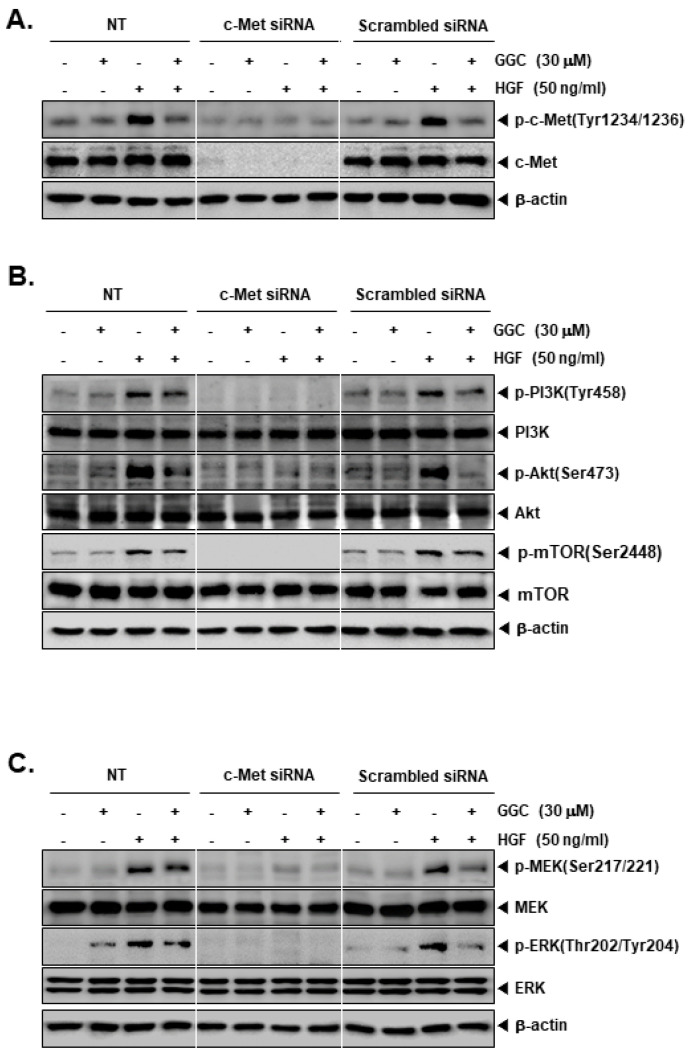
Effect of c-Met knock down on protein expression. HepG2 cells (5 × 10^4^ cells/well) were transfected with 100 nM c-Met siRNA or scrambled siRNA for 48 h. The transfected cells were pre-treated with GGC (30 μM) for 2 h, then HGF (50 ng/mL) treated for 30 min. Protein levels of various c-Met downstream signaling genes were measured by Western blotting. (**A**) Western blot analysis for p-c-Met (Tyr1234/1236) and c-Met. (**B**) Western blot analysis for p-PI3K (Tyr458), PI3K, p-Akt (Ser473), Akt, p-mTOR (Ser2448), and mTOR. (**C**) Western blot analysis for p-MEK (Ser217/221), MEK, p-ERK (Thr202/Tyr204), ERK, and β-actin. The results shown are representative data of three independent experiments.

We knockdown c-Met levels by c-Met siRNA transfection in HepG2 cells. Because the HGF acted as ligand for c-Met, it was hypothesized that HGF may not work upon the *c-Met* gene silencing. As shown, c-Met siRNA transfected cells had not impact on the expression of c-Met but non-treated and scrambled siRNA transfected cells displayed a substantial HGF-stimulated c-Met expression (Figure 3A). Therefore, HGF-induced phosphorylation of downstream signals (PI3K, Akt, mTOR, MEK, and ERK) also showed minimal expression in c-Met transfected cells (Figure 3B,C). On the contrary, control cells showed that GGC significantly down-regulated the phosphorylation of c-Met and downstream signals. However, c-Met inhibition had no effects on the total PI3K, Akt, mTOR, MEK, and ERK expression.

### 2.4. c-Met Gene silencing Upregulated GGC-induced Apoptosis

**Figure 4:**
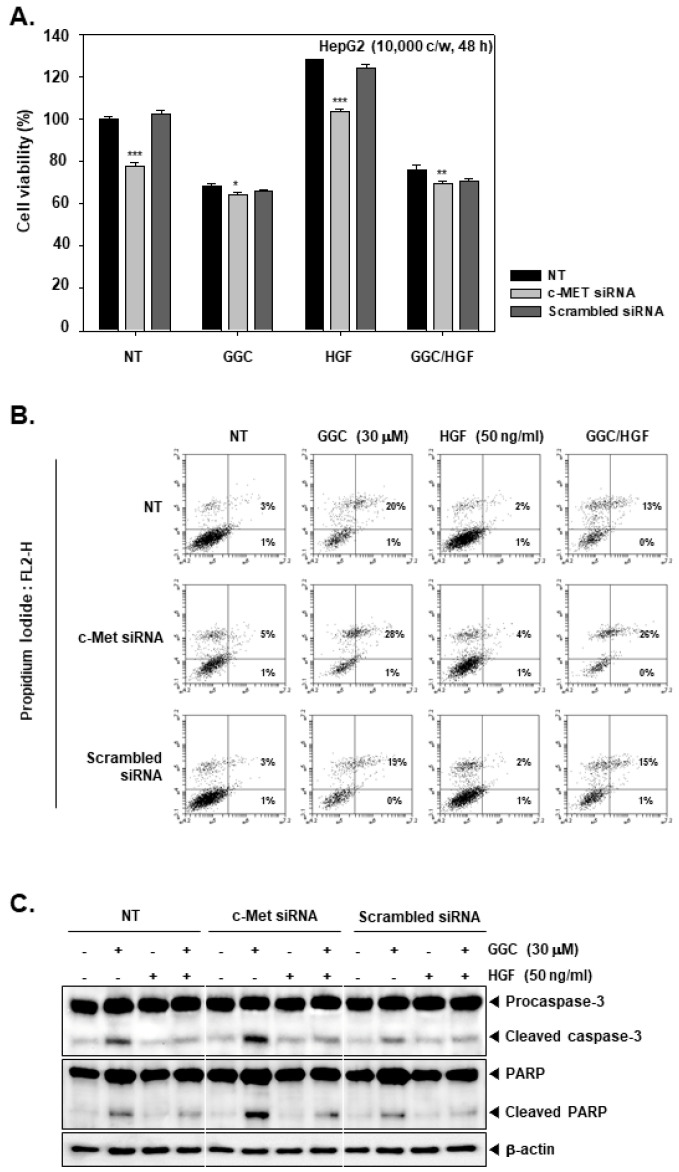
Effect of c-Met knock down on apoptosis. HepG2 cells (5 × 10^4^ cells/well) were transfected and treated as described in Figure 3. (**A**) Cell viability was therafter measured by MTT assay. The results are presented as the mean ± SD. * *p* <0.05, ** *p* <0.01, *** *p* <0.001 compared to the control. (**B**) Transfected cells were pre-treated with GGC (30 μM) for 2 h, then HGF (50 ng/mL) treated for 24 h. The cells were stained with Annexin V FITC as well as PI, and then analyzed by flow cytometry. (**C**) Transfected cells were pre-treated with GGC (30 μM) for 2 h, then HGF (50 ng/mL) treated for 24 h and western blotting was done. The results shown are representative data of three independent experiments.

HepG2 cells were transfected and re-seeded to compare the cell viability between c-Met siRNA transfected cells and control cells (Figure 4A). The cells were pre-treated with GGC (30 μM) for 2 h and treated with HGF (50 ng/mL) for total 48 h. c-Met transfected cells had lower cell viability than control cells. HGF increased the cell viability in only control groups and there were almost no changes on c-Met transfection group. However, GGC down-regulated the cell viability on c-Met transfection and control groups. We determined the apoptosis by Annexin V assay (Figure 4B). GGC increased the apoptosis and c-Met siRNA transfection cells had a higher percentage of late apoptosis than control cells. In addition, we examined the expression of Caspase-3 and PARP cleavage by Western blot analysis (Figure 4C). c-Met transfected cells had more activation of GGC-induced Caspase-3 and PARP cleavage.

### 2.5. GGC Inhibited Invasion and Migration

**Figure 5:**
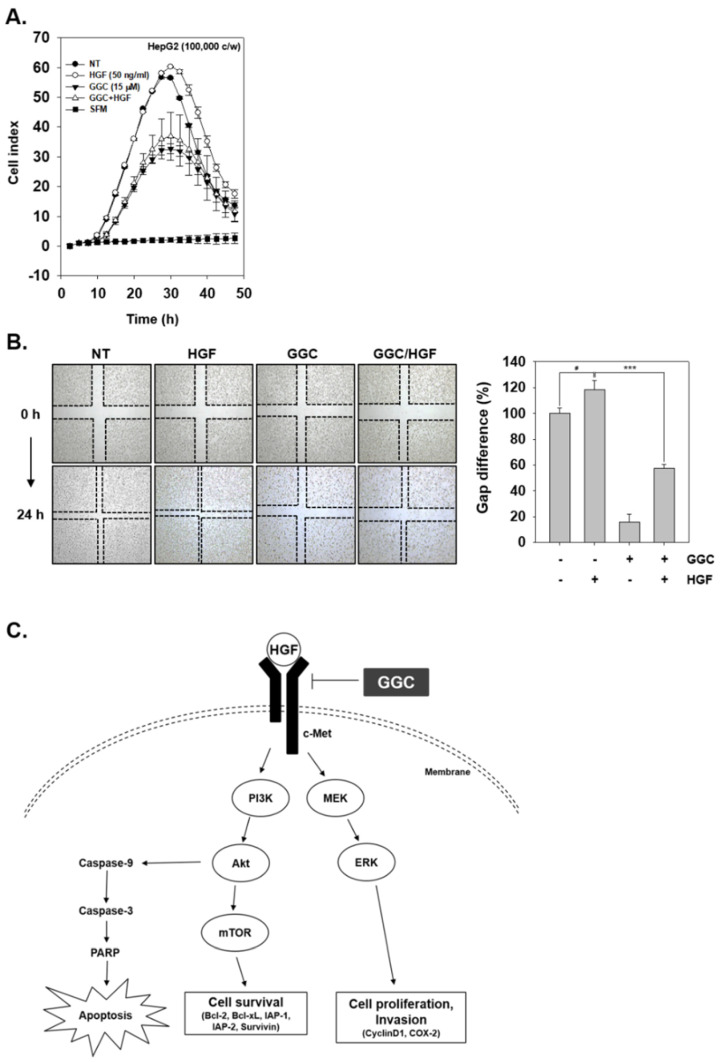
GGC inhibits invasion and migration. (**A**) HepG2 cells were treated with HGF (50 ng/mL), GGC (15 μM), or combination condition and invasion activity was determined by invasion assay. (**B**) Wound healing assay was used to determine migration. The width of wound was measured at time 0 and 24 h of incubation. Data represent means ± SD. # *p* < 0.05, *** *p* <0.001. (**C**) A schematic diagram showing the effects of GGC on c-Met signaling pathway.

We finally examined the role of GGC in abrogating HGF-induced invasion and migration. As shown in Figure 5A, GGC significantly suppressed tumor cell invasion activity. Moreover, HGF enhanced cell invasion, however, GGC down-regulated HGF-induced invasion as well. As shown in Figure 5B, the wound healing assay was examined with HGF, GGC, and the combination group. GGC attenuated the migration of HepG2 cells. Moreover, HGF-treated cells had less gap difference than non-treated cells, but GGC significantly mitigated HGF-induced migration.

## 3. Discussions

Although previous studies have demonstrated that GGC can display significant pharmacological activities, there are no previous studies about the possible anti-cancer effects of GGC and its mechanisms of actions against liver cancer cells [41,42,43]. In this study, we have elegantly demonstrated that GGC exerted a substantial anti-neoplastic activities through modulating HGF/c-Met signaling pathway in HCC cells. We found that GGC suppressed cell proliferation and induced apoptosis via down-regulating HGF/c-Met signaling cascades. Moreover, GGC inhibited invasion and migration in HepG2 cells and thus affected various hallmarks of tumor growth.

The aberrant activation of c-Met has been closely linked with tumor metastasis in HCC [24]. In addition, c-Met has been reported to activate a number of oncogenic signaling pathways such as Ras, MAPK, PI3K, Akt, and STAT3 [15,25,44,45,46]. These signaling pathways are known to regulate growth and metastasis in tumor models, including HCC [27]. Thus, the inhibition of c-Met activation may be a novel strategy to target HCC. We found that GGC suppressed c-Met signaling cascade activation in liver cancer cells. HGF induced activation of multiple c-Met downstream signaling cascades such as PI3K, Akt, mTOR, MEK, and ERK. GGC effectively abrogated the activation of all these oncogenic pathways. In addition, silencing of c-Met blocked the activation of c-Met as well as downstream pathways and the effect of GGC on HGF-induced c-Met phosphorylation was dramatically neutralized. However, analysis of the detailed mechanism(s) by which GGC can affect c-Met phosphorylation requires further experiments. For instance, it can be investigated if GGC can directly bind to HGF/c-Met axis or can affect c-Met kinase activity using the purified recombinant c-Met protein in future studies.

Akt can be activated by PI3K, and can promote tumorigenesis in tumor cells [47,48,49]. MEK activation can transduce signals through actively phosphorylating MAPK to promote cell proliferation [50]. We also demonstrated that GGC attenuated proliferation and caused apoptosis in HCC cells. We also found that GGC induced apoptosis via the cleavage of caspase-9, caspase-3, and PARP, whereas HGF treatment could substantially reduce the activation of these pro-apoptotic proteins. In addition, GGC reduced the expression of HGF-induced anti-apoptotic proteins in HCC cells. Several reports have demonstrated that the knockdown of c-Met can abrogate proliferation, tumorigenicity, and promote apoptosis [47,51,52,53]. Moreover, our results also showed that the deletion of c-Met can decrease cell viability and substantially augment GGC-induced apoptosis. Specifically, we found that GGC-induced apoptosis was increased in HepG2 cells transfected with c-Met si-RNA, thereby indicating that HepG2 cells transfected with c-Met si-RNA were more sensitive to GGC treatment. These results suggested that c-Met plays an important role in the sensitivity of HepG2 cells to the pro-apoptotic effects of GGC.

When HGF can bind to c-Met, several complex signaling cascades can be simultaneously induced and influence the processes of cell invasion and migration [54]. PI3K and MAPK, which are two key c-Met downstream signaling molecules, can function to control regulate growth, migration and invasion [50,55]. We demonstrated the GGC exposure significantly reduced invasion and migration in HGF-stimulated cells. Interestingly, GGC suppressed tumor cell invasion and migration whereas, HGF treatment mitigated GGC-induced invasion and migration.

Overall, our findings indicate that GGC can exerts anti-tumor effects through the effective attenuation of c-Met signaling cascades in HCC cells. Although further studies are required to determine whether GGC can be employed along with existing treatment modalities for HCC management, our results demonstrate a promising therapeutic activity of GGC against HCC.

## 4. Materials and Methods

### 4.1. Reagents

Ginkgolide C was purchased from Weikeqi Biological Technology (Chengdu, Sichuan, China). 3-(4,5-dimethylthiazol-2-yl)-2,5-diphenyltetrazolium bromide (MTT) and bovine serum albumin (BSA) were purchased from Sigma-Aldrich (St. Louis, MO, USA). Alexa Fluor^®^ 488 donkey anti-rabbit IgG (H + L) antibody was obtained from Life Technologies (Grand Island, NY, USA). Hepatocyte growth factor (HGF) was purchased from PeproTech (Offenbach, Germany). FITC Annexin V Apoptosis Detection Kit was purchased from BD Pharmingen™ (BD Biosciences, Becton-Dickinson, Franklin Lakes, NJ, USA). iN-fect™ in vitro Transfection Reagent was obtained from iNtRON Biotechnology (Seongnam, Korea). Anti-phospho-c-Met, anti-c-Met, anti-phospho-PI3K(Tyr458), anti-PI3K, anti-phospho-Akt(Ser473), anti-phospho-mTOR(Ser2448), anti-mTOR, anti-phospho-MEK(Ser217/221), anti-MEK, anti-phospho-ERK, anti-ERK, anti-caspase-9, anti-cleaved-caspase-9, and anti-cleaved-caspase-3 antibodies were purchased from Cell Signaling Technology (Beverly, MA, USA). Anti-Akt, anti-caspase-3, anti-PARP, anti-Bcl-2, anti-Bcl-xL, anti-Survivin, anti-IAP-1, anti-IAP-2, anti-Cyclin D1, anti-COX-2, and anti-β-actin antibodies were purchased from Santa Cruz Biotechnology (Santa Cruz, CA, USA).

### 4.2. Cell Lines and Culture Conditions

Human hepatocellular carcinoma HepG2 cells were obtained from American Type Culture Collection (Manassas, VA, USA). HepG2 cells were cultured in RPMI 1640 medium containing 10% fetal bovine serum (FBS) and 1% penicillin-streptomycin. In addition, cells were maintained at 37 °C under 5% CO_2_ atmosphere.

### 4.3. MTT Assay

Cell viability was measured using an MTT assay as described previously [56]. Cell viability was normalized as relative percentage in comparison with non-treated controls.

### 4.4. Western Blot Analysis

For the detection of various antibodies, GGC and HGF-treated whole-cell extracts were lysed in a lysis buffer and Western blotting was carried out as elaborated previously [57]. The membranes were detected using a chemiluminescence (ECL) (EZ-Western Lumi Femto, DOGEN).

### 4.5. Immunocytochemistry

HepG2 cells were seeded in 8-well chamber slide, and immunohistochemistry was done as explained previously [58]. Then, the fluorescence signal was analyzed by Olympus FluoView FV1000 confocal microscope (Tokyo, Japan).

### 4.6. Cell Transfection and siRNA Knockdown

The iN-fect™ in vitro Transfection Reagent (iNtRON Biotechnology, Seongnam, Korea) was used for the transfection for RNA interference to knock down c-Met expression. HepG2 cells (5 × 10^4^ cells/well) were transfected with c-Met siRNA (100 nM) or scrambled siRNA (100 nM) (SN-1002; BIONEER, Daejeon, Korea) for 48 h in serum-free media. After transfection, cells were pre-treated with GGC (30 μM) for 2 h and treated with HGF (50 ng/mL).

### 4.7. Cell Cycle Analysis

HepG2 cells were treated with GGC (0, 5, 15, and 30 μM) for 24 h. After that, cells were collected and fixed by EtOH for overnight. Then, cells were incubated with RNase A for 1 h at 37 °C and stained with propidium iodide. Cells were analyzed by BD Accuri™ C6 Plus Flow Cytometer (BD Biosciences, Becton-Dickinson, Franklin Lakes, NJ, USA) with BD Accuri C6 Plus software.

### 4.8. Annexin V Assay

Annexin V assay was performed to determine apoptosis as described earlier [59].

### 4.9. TUNEL Assay

HepG2 cells were treated with GGC (0, 5, 15, and 30 μM) for 24 h and thereafter TUNEL staining was performed as elaborated previously [60].

### 4.10. Real-time Cell Proliferation Analysis

Cell growth behavior was performed using xCELLigence Real-Time Cell Analyzer (RTCA) DP instrument (Roche Diagnostics GmbH, Mannheim, Germany) as described previously [61].

The real-time monitoring of HepG2 cells invasion was performed using xCELLigence Real-Time Cell Analyzer (RTCA) DP instrument (Roche Diagnostics GmbH, Germany) as described before [62].

### 4.11. Wound Healing Assay

Wound healing assay was performed in monolayer of HCC cells as described before [63]. Width of wound was observed by using a microscope (Nikon ECLIPSE Ts2, Nikon Corporation, Tokyo, Japan) at time 0 and 24 h. The gap distance of the wound was measured at four different sites and non-treated samples were used as controls.

### 4.12. Statistical Analysis

All experiments are presented as the mean ± standard deviation (SD). The statistical significance of the data compared with the non-treated control was determined using the Mann–Whitney U test. Significance was set at *p* < 0.05.
